# Modeling Superconducting Critical Temperature of 122-Iron-Based Pnictide Intermetallic Superconductor Using a Hybrid Intelligent Computational Method

**DOI:** 10.3390/ma14164604

**Published:** 2021-08-16

**Authors:** Oluwatobi Akomolafe, Taoreed O. Owolabi, Mohd Amiruddin Abd Rahman, Mohd Mustafa Awang Kechik, Mohd Najib Mohd Yasin, Miloud Souiyah

**Affiliations:** 1 Physics and Electronics Department, Adekunle Ajasin University, Akungba Akoko 342111, Nigeria; oluwatobiakomolafe13@gmail.com (O.A.); owolabitaoreedolakunle@gmail.com (T.O.O.); 2 Department of Physics, Faculty of Science, Universiti Putra Malaysia, Serdang 43400, Malaysia; mmak@upm.edu.my; 3 Advanced Communication Engineering (ACE), Centre of Excellence, Universiti Malaysia Perlis, Kangar 01000, Malaysia; 4 Department of Mechanical Engineering, College of Engineering, University of Hafr Al Batin, P.O. Box 1803, Hafr Al Batin 31991, Saudi Arabia; souiyah@uhb.edu.sa

**Keywords:** 122-iron-based superconductor, structural transformation, support vector regression, ionic radii, particle swarm optimization, lattice parameters

## Abstract

Structural transformation and magnetic ordering interplays for emergence as well as suppression of superconductivity in 122-iron-based superconducting materials. Electron and hole doping play a vital role in structural transition and magnetism suppression and ultimately enhance the room pressure superconducting critical temperature of the compound. This work models the superconducting critical temperature of 122-iron-based superconductor using tetragonal to orthorhombic lattice (LAT) structural transformation during low-temperature cooling and ionic radii of the dopants as descriptors through hybridization of support vector regression (SVR) intelligent algorithm with particle swarm (PS) parameter optimization method. The developed PS-SVR-RAD model, which utilizes ionic radii (RAD) and the concentrations of dopants as descriptors, shows better performance over the developed PS-SVR-LAT model that employs lattice parameters emanated from structural transformation as descriptors. Using the root mean square error (RMSE), coefficient of correlation (CC) and mean absolute error as performance measuring criteria, the developed PS-SVR-RAD model performs better than the PS-SVR-LAT model with performance improvement of 15.28, 7.62 and 72.12%, on the basis of RMSE, CC and Mean Absolute Error (MAE), respectively. Among the merits of the developed PS-SVR-RAD model over the PS-SVR-LAT model is the possibility of electrons and holes doping from four different dopants, better performance and ease of model development at relatively low cost since the descriptors are easily fetched ionic radii. The developed intelligent models in this work would definitely facilitate quick and precise determination of critical transition temperature of 122-iron-based superconductor for desired applications at low cost with experimental stress circumvention.

## 1. Introduction

The discovery of superconducting property in iron-based materials has significantly facilitated the exploitation of new classes of superconductors for commercial applications, especially in tapes and wires fabrications [1]. Among the classes of iron-based superconductors, 122 type with structural formula AFe_2_As_2_ (where A represents “alkali” or “alkali earth metal”) demonstrates fascinating and attractive characteristic features such as ultra-high upper critical fields, high superconducting critical temperature and low anisotropy [2,3]. These aforementioned unique properties render the superconductor indispensable for applications where a high magnetic field is needed [4]. Iron-based family (AFe_2_Se_2_), where the toxic arsenic is replaced with environmentally friendly selenium, also falls within 122 families of iron-based superconductors [5]. The fact that the 122 family of iron-based superconductors serves as a competition playground between superconductivity and spin density wave with itinerant 3D magnetic ordering (covering incommensurate and longitudinal) contributes immensely to the uniqueness of this class of material [6]. A single crystal of this family can be easily prepared with a high level of purity without the formation of multiple phases, and this has contributed immensely to the in-depth study of its physical properties. Spin density wave with a characteristic lattice distortion from tetragonal symmetry to orthorhombic is often associated with the parent compounds of the 122 superconducting family. Antiferromagnetic ordering is observed at lower temperatures in this family (AFe_2_As_2_, where A = rare earth element) of iron-based superconductor with 4F magnetic moments and a characteristic non-zero localization. Magnetic moment (4F) re-orientation occurs with 4F ferromagnetic component formation as a result of doping (through which superconductivity emerges) when the superconducting material houses 4F antiferromagnetic ordering [7]. Therefore, superconductivity appears after applying external pressure or through doping in which the spin density wave order is suppressed. In order to achieve room pressure superconductor, hole doping, electron doping and iso-electronic substitutions are possible [8]. Iso-electronic substitution involves the replacement of arsenic by other elements such as phosphorus or the application of strong perturbation to Fe-As layer through perturbation of Fe environment since pnictogen tetrahedrally coordinates the Fe atoms. The observed structural distortions after doping as well as the ionic radii of the incorporated dopants are explored in this present work to model superconducting critical temperature of the compound using hybrid support vector regression and particle swarm optimization method.

The electronic structure that is close to Fermi energy controls the close proximity between phase transition (magnetic and structural) and superconductivity. A surface within momentum space that occupies all the fermionic states with momentum less than the Fermi momentum is referred to as the Fermi surface. Iron d-orbital are the occupants of iron-based superconductor Fermi surface, which make the surface very sensitive to doping, temperature and pressure [9]. The 122 iron-based superconducting compounds crystalize at room temperature into tetragonal I4/mmm-139 space group while cooling to lower temperature, resulting in structural transformation from tetragonal to orthorhombic structure, and the unit cell characterizing the low-temperature phase enjoys 45° rotation in the x–y plane with large basal cell edges. Magnetic transition often follows the observed structural transformation. The structural transition is driven by the orbital ordering, which, in turn, induces anisotropy in the parent magnetism and ultimately triggers the magnetic transition. Structural transformation to Fmmm orthorhombic structure takes place during low-temperature cooling, which controls the emergence of superconductivity in the compound. The superconducting critical temperature of 122-iron-based superconductors attains its highest value when the magnetism in the host compound is strongly hindered or totally destroyed. Destruction of magnetism is induced through high-pressure application and hole or electron doping. The hole-doping that ultimately increases FeAs positive charges further suppresses magnetism for the emergence of superconductivity. Superconductivity is favored in these materials with magnetic ground destabilization. The magnetic interactions which drive the magnetic ordering produce electron-pairing interaction that serves as the background of superconductivity. Therefore, co-existence between magnetism and superconductivity often arises in doped samples. Sustenance of room temperature tetragonality at low-temperature scenarios sometimes promotes superconductivity emergence. This sustenance is achieved through foreign material incorporation into the parent compound. Ionic radii (RAD) of the incorporated dopants as well as the concentration of each of the dopants serve as the descriptors for developing the particle-swarm-based support vector regression PS-SVR-RAD model while the lattice parameters of the doped samples are employed in developing the PS-SVR-RAD model for estimating the superconducting critical temperature of the doped samples.

Support vector regression belongs to a class of machine learning intelligent algorithms which conveniently, effectively and efficiently minimize generalized error bound using structural risk minimization principle [10,11]. The algorithm employs a kernel trick for data mapping and transformation to feature space where the regression problem is precisely addressed. The initial definition of error threshold called “epsilon” and the possible inclusion of non-zero slack variables enable the algorithm to address non-linear regression problems with close proximity between the measured and estimated target. As such, real-life applications of the SVR algorithm cuts across many fields of study [12,13,14,15,16,17,18,19]. The algorithm parameters such as the defined error threshold epsilon, penalty factor and mapping function parameter are very germane to the successful acquisition of patterns connecting the desired model target with the descriptors. An evolutionary algorithm with swarm operational principle is employed for the selection of optimal combinatory choice of these parameters. The outstanding features of the employed particle swarm optimization algorithm include fast convergence, difficulty in trapping inside local solutions and evasion of premature convergence.

The rest of the manuscript is organized in the following structure: Section 2 discusses the mathematical foundation of the support vector regression and particle swarm optimization algorithms, while Section 3 presents the physical description and acquisition of the dataset employed for computation. The computational details of the developed hybrid model are also presented in Section 3. Section 4 presents the results of the developed models. A comparison of the performance of PS-SVR-RAD and PS-SVR-LAT models is presented in section four. Section 5 presents the conclusions.

## 2. Mathematical Details of the Hybridized Algorithms

The mathematical explanations of the support vector regression algorithm and that of the particle swarm optimization algorithm are contained in this section.

### 2.1. Mathematical Background of the Support Vector Regression

The primary objective of the support vector regression (SVR) algorithm is to acquire the pattern and function connecting the descriptors with the target [20]. Consider 122-iron-based superconductors doped with external materials while the distorted lattice parameter (for the case of PS-SVR-LAT), ionic radii as well as dopant concentration (in the case of PS-SVR-RAD) and superconducting temperature ($T_{j}$) are expressed as $\left\{(\varphi_{0},T_{0}),\ldots ,(\varphi_{j},T_{j})\right\}$, where $\varphi_{j}$ represents the descriptors. The algorithm aims at establishing a relationship shown in Equation (1) [21].

(1) T(φ)=ω×ϑ(φ)+c

where the vector weight, bias and the mapping function are, respectively, represented as ω, c and ϑ(φ). SVR algorithm minimizes the Euclidean norm ${\left\|\omega \right\|}^{2}$ and implements a parameter called penalty parameter for penalizing and regularizing distortion (ionic radii in the case of PS-SVR-RAD model) in 122-iron-based superconducting data outside the loss zone purposely to reduce the complexity of the final model. Equation (2) presents the modification of the optimization problem while the governing constraints are expressed in Equation (3) [13,22].

(2) $$Min\;E=\frac{{\left\|\omega \right\|}^{2}}{2}+\alpha \sum_{j=1}^{r}\left(\delta_{j}+\delta_{j}^{*}\right)$$

(3) $$(\begin{array}{c} t_{j}-\omega \times \vartheta (\varphi_{j})-c\leq \varepsilon +\delta_{j}\\ \omega \times \vartheta (\varphi_{j})+c-t_{j}\leq \varepsilon +\delta_{j}^{*}\\ \delta_{j}^{*}\geq 0,\delta_{j}\geq 0 \end{array}$$

The expression presented in Equation (3) considers a situation whereby the objective of the SVR algorithm in Euclidean minimization becomes difficult due to other external constraints. Slack variables ($\delta_{j}^{*}$ and $\delta_{j}$) maintain the minimization principle of model Euclidean norm. The penalty parameter α strikes a balance between model complexity and error minimization below the threshold value represented as epsilon ε [15]. The experimentally measured superconducting critical temperature for 122-iron-based superconductors for all the considered doped compounds is represented as $t_{j}$ in Equation (3). In an attempt to solve the optimization problem through Lagrange formalisms, multipliers ($\psi^{*}$ and ψ) are introduced. The optimization problem is transformed as presented in Equation (4) with the expression presented in Equation (5) as constraints [23].

(4) $$\begin{array}{l} -\frac{1}{2}\sum_{j,i}^{z}\left(\psi_{j}-\psi_{j}^{*}\right)\left(\psi_{i}-\psi_{i}^{*}\right)\vartheta (\varphi_{j},\varphi_{i})-\sum_{j}^{z}\psi_{j}\left(t_{j}+\varepsilon \right)\\ +\sum_{j}^{z}\psi_{j}^{*}\left(t_{j}-\varepsilon \right)=0 \end{array}$$

(5) $$\{\begin{array}{c} \sum_{j}^{z}\left(\psi_{j}-\psi_{j}^{*}\right)=0\\ 0\leq \psi_{j},\psi_{j}^{*}\leq \alpha ,j=1,2,\ldots ,z \end{array}$$

If the optimal solutions of the convex optimization problem are assumed to be $\psi =\left[\psi_{1},\psi_{2},\ldots \ldots ,\psi_{z}\right]$ and $\psi^{*}=\left[\psi_{1}^{*},\psi_{2}^{*},\ldots \ldots ,\psi_{z}^{*}\right]$, then the weight vector and the bias can be computed and presented as shown in Equations (6) and (7), respectively.

(6) $$\omega^{*}=\sum_{j}^{z}\left(\psi_{j}-\psi_{j}^{*}\right)\vartheta (\varphi_{j})$$

(7) $$\begin{array}{l} c^{*}=\frac{1}{s_{v}}\sum_{0<\psi_{j}<\alpha }\left(t_{j}-\sum_{\varphi_{j}\in S_{v}}(\psi_{j}-\psi_{j}^{*})\vartheta (\varphi_{j},\varphi_{i})-\varepsilon \right)\\ +\frac{1}{s_{v}}\sum_{0<\psi_{j}<\alpha }\left(t_{j}-\sum_{\varphi_{i}\in S_{v}}(\psi_{i}-\psi_{i}^{*})\vartheta (\varphi_{j},\varphi_{i})-\varepsilon \right) \end{array}$$

where the number of acquired support vectors during model development is represented as $s_{v}$. Equation (8) presents the final regression function, where the expression for the implemented non-linear mapping function is shown in Equation (9).

(8) $$T(\varphi )=\sum_{j=1}^{z}\left(\psi_{j}-\psi_{j}^{*}\right)\vartheta (\varphi_{j},\varphi )+c^{*}$$

(9) $$\vartheta (\varphi_{j},\varphi_{i})=\exp\left(-\lambda {\left\|\varphi_{j}-\varphi_{i}\right\|}^{2}\right)$$

where λ = kernel parameter.

The kernel parameter (λ), penalty parameter (α) and the error threshold (ε) contribute immensely to the precision and accuracy of the model. These parameters are fine-tuned in a combinatory form in this work using the particle swarm optimization approach.

### 2.2. Description of the Particle Swarm Optimization Method

The particle swarm optimization (PSO) algorithm is a stochastic population-based evolutionary optimization method developed by Eberhart and Kennedy in 1995 [24]. The social-behavioral pattern of the swarm combined with their intelligence forms the basis of this algorithm [25]. The particles within the swarm navigate and transverse the search space for objective function evaluation. Particles in the swarm are identified by their previous as well as current positions and velocities [26]. The main stages of the algorithm operational principles include particle fitness evaluation, updating the global as well as individual best and updating the velocity and position of every particle [27]. Equations (10) and (11) are implemented while updating the velocity and position of swarm particles [10,28].

(10) $$v_{m}(k+1)=\eta v_{m}(k)+\mu_{1}\sigma_{1}\left(\varsigma_{m}(k)-x_{m}(k)\right)+\mu_{2}\sigma_{2}\left(\gamma_{m}(k)-x_{m}(k)\right)$$

(11) $$x_{m}(k+1)=x_{m}(k)+v_{m}(k+1)$$

where *m* is the number of particle, $v_{m}(k)$ = velocity of mth particle in iteration period k, $x_{m}(k)$ = position of mth particle in iteration period k, η = coefficient of inertial weight, $\mu_{1}$ = coefficient of personal learning, $\mu_{2}$ = coefficient of global learning, $\sigma_{1}$ = selected random number within the range of 0–1, $\sigma_{2}$ = selected random number within the range of 0–1, $\gamma_{m}(k)$ = best position of the swarm in iteration period k and $\varsigma_{m}(k)$ = individual best position in iteration period k.

## 3. Computational Presentation and Methodology of the Developed Hybrid Model

The employed computational strategies for algorithm hybridization are presented in this section. Acquisition and physical description of the dataset are also contained in this section. The section further presents a stoichiometric formulation of the 122 family of superconductors that can be modeled using the developed PS-SVR-RAD model.

### 3.1. Description of the Dataset and Stoichiometric Expression of the Compounds That Are Well Captured within the PS-SVR-RAD Model

Structural lattice parameters that serve as the descriptors to the developed PS-SVR-LAT model and their corresponding superconducting critical temperature are extracted from the literature [29,30,31]. The descriptors of the developed PS-SVR-RAD model are the ionic radii and the concentrations of the dopants. An experimental dataset from 21 compounds of 122-iron-based superconductors was employed in building the PS-SVR-LAT model, while the development of the PS-SVR-RAD model utilizes 44 data points extracted from distorted 122-iron-based superconductors. Equation (12) presents the stoichiometric expression of the 122 family of iron-based superconductors whose superconducting critical temperature can be estimated by the developed PS-SVR-RAD model.

(12) $$PS-SVR-RAD=D_{1-\alpha }A_{\alpha }Fe_{2-\beta }B_{\beta }\psi_{2-\gamma }C_{\gamma }$$

where A = alkali or alkali earth metal, D = foreign element attached to alkali or alkali earth metal, α = concentration of D dopant. B = foreign element attached to iron as dopant, β = concentration of B, ψ = arsenic or selenium, C = foreign element attached to arsenic or selenium as dopant and γ = concentration of C. Each of the investigated 122 family of iron-based superconductors used for modeling and simulation is subjected to the stoichiometric description presented in Equation (12). The uniqueness of this expression is that it allows the incorporation of four different dopants into the parent 122 type of pnictide, while the superconducting critical temperature is estimated by the developed PS-SVR-RAD model. During descriptors extraction for implementation using the PS-SVR-RAD model, dopants that are absent within the description of Equation (12) are assigned a zero value. For example, during extraction of modeling data for the Ba_0.67_K_0.33_Fe_2_As_2_ 122-iron-based superconductor, D is the ionic radius of barium (Ba) since the atoms of potassium are replaced with that of barium, A is the ionic radius of potassium (K), α = 0.33, B = β = 0, ψ represents the ionic radius of arsenic and C = γ = 0. It should be noted that the effective ionic radii of the constituent elements with diverse ionic charges and spins were employed as descriptors to the developed PS-SVR-RAD model due to the existence of a non-linear relationship between them and the superconducting transition temperature, as revealed from the conducted preliminary statistical analysis. The employed effective ionic radii in picometer (pm) and their corresponding elements are summarized as (138, 50, 152, 167, 150, 135, 58, 184, 60, 65, 103.2) = (K, Se, Rb, Cs, Tl, Ba, As, S, Ni, Co, La). The comprehensive details of the employed dataset for the developed models are presented in the Supplementary Materials section of the manuscript.

### 3.2. Computational Strategies Employed for Hybrid Model Development

The development of PS-SVR-LAT and PS-SVR-RAD, with which superconducting critical temperatures of 122-iron-based superconductors are estimated, was conducted within a MATLAB computing environment environment (MATLAB, 2015a, 2015, MathWorks, Natick, MA, USA). The essence of a particle swarm optimization algorithm inclusion in support vector regression algorithm is to optimize the parameters influencing the performance SVR in a combinatory manner so the precision, robustness and accuracy of the hybrid algorithm can be enhanced. The available ionic radii and the concentration of the dopants for developing the PS-SVR-RAD model are randomized initially to ensure uniform data distribution within the training and testing data subset. Similar randomization was conducted for the proposed PS-SVR-LAT model, while the crystal lattice parameters of doped 122-iron-based superconductors are the descriptors in this case. Data separation and division in the ratio of 1:4 follow immediately after randomization procedures for testing and training samples, respectively. This indicates that 17 data points and 4 data points are, respectively, available for the training and testing stage of PS-SVR-LAT model development, while 36 and 8 data points are the separated training and testing set of data for the case of the PS-SVR-RAD model. The training set of data helps in support vectors acquisition and generation, while the efficacy and precision of the model are assessed using a testing set of data. The implemented test-set cross-validation technique in this work is due to the limited data characterizing this area of research. Procedures for optimization algorithm hybridization with support vector regression are itemized as follows.

Step I: Initialization of optimization algorithm parameters: the user-defined parameters in PSO such as the maximum iteration number ($k_{\max}$), size of the swarm population ($P_{s}$) coefficient of personal learning ($\mu_{1}$), coefficient of global learning $\mu_{2}$, probable solution search space and inertial weight are initiated. For the PS-SVR-RAD model, the upper search spaces for the probable hyper-parameter solutions are 1000, 0.009 and 0.009 for the penalty factor, error threshold epsilon and kernel parameter for the Gaussian mapping function, respectively. The lower search spaces for the same order of hyper-parameter are defined as 1, 0.0001 and 0.0001. The upper search spaces of the developed PS-SVR-LAT model are defined as 500, 0.8 and 0.8, while the lower bounds of the search space are defined as 1, 0.1 and 0.1 for the penalty factor, error threshold epsilon and kernel parameter for the Gaussian mapping function, respectively. It is worth mentioning that a random initial search was conducted before the choice of upper and lower limits of search spaces.

Step II: Random initialization of the swarm velocity and position: the potential solutions of the optimization problems as represented by swarm positions and velocities are randomly generated within the limits of the search spaces. These velocities and positions are subsequently updated from generation to generation until global solutions are attained. 

Step III: Fitness of the particle in swarms: the particle fitness is evaluated through the development of SVR based model while the root mean square error (RMSE) of the estimated superconducting critical temperature of the testing set (TS) of data serves as the fitness of the particle. The implemented procedures for SVR algorithm development for each particle are as follows; (a) mapping function selection from Gaussian, sigmoid and polynomial function (b) training of SVR algorithm with mth particle, training dataset and the selected mapping function in Step a. (c) Support vectors generated and acquired at Step b are subjected to validation and evaluation using a testing dataset through RMSE computation. (d) The trained SVR algorithm is evaluated using RMSE obtained from Step c. (e) Step b to Step d are repeated for other particles in the swarm, while the best particle position (as represented by the lowest RMSE of the testing set of data) is recorded and saved as $\varsigma_{m}(k)$. (f) Repeat Step a to Step e for other mapping functions, while the best particle position as represented by the lowest testing data RMSE for all the investigated mapping functions is recoded and saved as $\gamma_{m}(k)$.

Step IV: Updating the position of individual best if necessary: if $\varsigma_{current}>\varsigma_{m}$, update as $\varsigma_{current}=\varsigma_{m}$. Proceed to the next step if otherwise. $\varsigma_{current}$ represents the current position.

Step V: Updating the global position if necessary: if $\varsigma_{current}>\gamma_{m}$, then update as $\varsigma_{current}=\gamma_{m}$. Proceed to the next step if otherwise.

Step VI: Check for the maximum number of particles: if the indexed particle is greater than the defined maximum number of particles at the commencement of the optimization process, proceed to the next step. Go back to Step III if otherwise.

Step VII: Global best position for fitness function evaluation: with $\gamma_{m}$, compute the fitness function of the particles.

Step VIII: Updating the velocity and position: the positions of the particles are updated using Equation (11), while Equation (10) updates the velocity.

**Figure 1:**
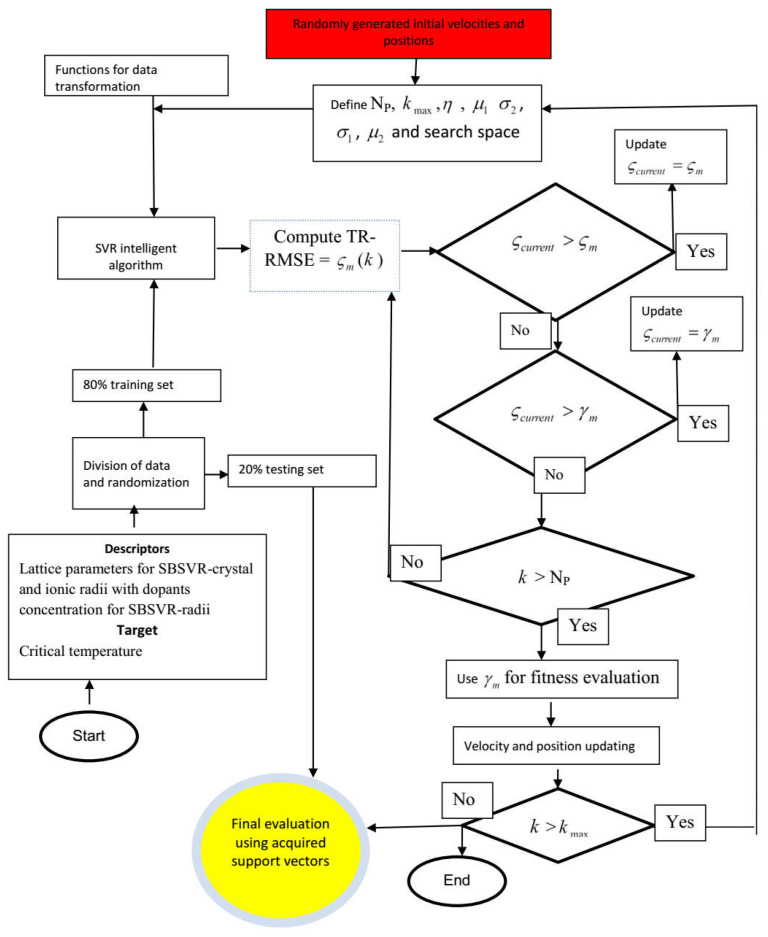
Computational chart of the developed PS-SVR model. (PS = Particle swarm, SVR = support vector regression, RAD = radii, LAT = lattice, TR = training, RMSE = root mean square error, Np = number of population).

Step IX: Stopping criteria: the entire cycles are repeated continuously, and the algorithm is brought to stop if 50 consecutive iterations converge to the same RMSE value for the testing dataset or the defined maximum iteration number is achieved. Figure 1 presents a comprehensive flow chart of the developed PS-SVR-RAD and PS-SVR-LAT models.

## 4. Results and Discussion

This section presents the outcomes of the developed PS-SVR-LAT and PS-SVR-RAD models. The dependence of the convergence of the developed models with the size of the populations is investigated. A comparison of the performances of the developed models is presented. 

### 4.1. Hybrid Model Convergence with the Population Size

**Figure 2:**
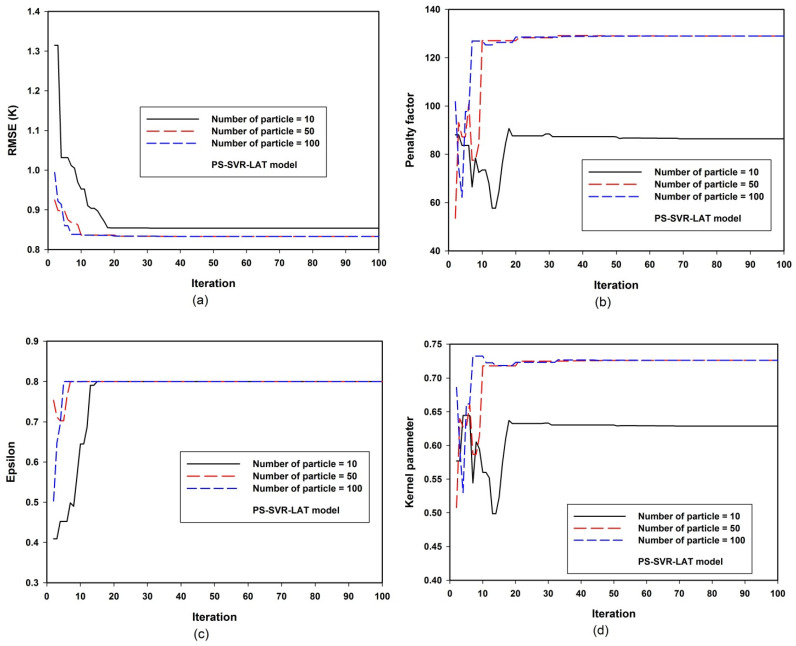
Convergence and PS-SVR-LAT model sensitivity to hyper-parameters. (**a**) PS-SVR-LAT model sensitivity to root mean square error(RMSE) at various population size (**b**) PS-SVR-LAT model sensitivity to penalty factor at various population size (**c**) PS-SVR-LAT model sensitivity to epsilon at various population size (**d**) PS-SVR-LAT model sensitivity to kernel parameter at various population size.

**Figure 3:**
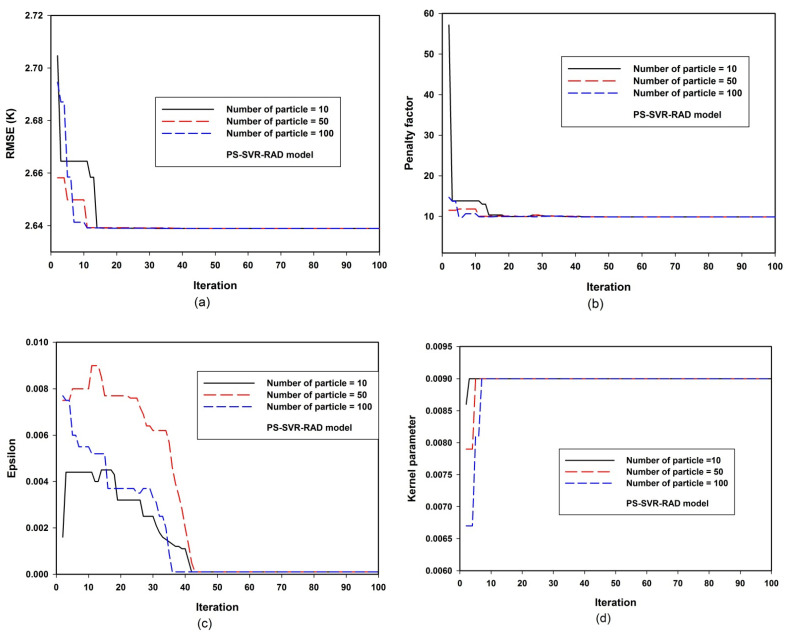
Convergence and PS-SVR-RAD model sensitivity to hyper-parameters. (**a**) PS-SVR-RAD model sensitivity to root mean square error(RMSE) at various population size (**b**) PS-SVR-RAD model sensitivity to penalty factor at various population size (**c**) PS-SVR-RAD model sensitivity to epsilon at various population size (**d**) PS-SVR-RAD model sensitivity to kernel parameter at various population size.

The sensitivities of the developed PS-SVR-LAT and PS-SVR-RAD models to hyper-parameters are presented in Figure 2 and Figure 3, respectively. Figure 2a presents the convergence of RMSE between measured and estimated superconducting critical temperature for the testing set of data. The presented convergence depends on the size of the swarm population, while local convergence was observed with the exploration of the search space by 10 particles. Optimum convergence was attained with a population size of 50, while a further increase in population size beyond this optimum value has no global solution convergence significance. The variation of penalty factor with population size is presented in Figure 2b for the developed PS-SVR-LAT model that employs lattice parameters of iron-based superconductor as descriptors. The presence of 10 particles within the search space leaves the convergence of the penalty factor to a lower value. Optimum convergence was attained with a population size of 50, as presented in Figure 2b. The error threshold epsilon for the developed PS-SVR-LAT model investigated at different population sizes is presented in Figure 2c, while the convergence of this parameter does not depend on the population size. A similar value of convergence is attained using different numbers of particles in the search space.

Figure 2d depicts the variation of the value of the mapping function parameter with the particle population size. A total of 10 particles exploring the defined search space converge at a lower value, while global convergence was achieved with a particle population size of 50 and 100. For the sensitivity and convergence of the developed PS-SVR-RAD model, Figure 3 presents the model convergence and sensitivity variations as a function of the population size. Figure 3a shows the error convergence of the model. The obtained results, as presented in the figure, show that the model convergence does not change with changes in the size of the particle population. This confirms the robustness of the developed PS-SVR-RAD model. Similar convergence was achieved with penalty factor convergence, epsilon sensitivity convergence and kernel parameter sensitivity convergence, as presented in Figure 3b–d, respectively. The values of each of the parameters as obtained from PSO are presented in Table 1 for the developed PS-SVR-LAT and PS-SVR-RAD models.

### 4.2. Evaluation and Comparison of the Performance of the Developed Hybrid Models 

**Figure 4:**
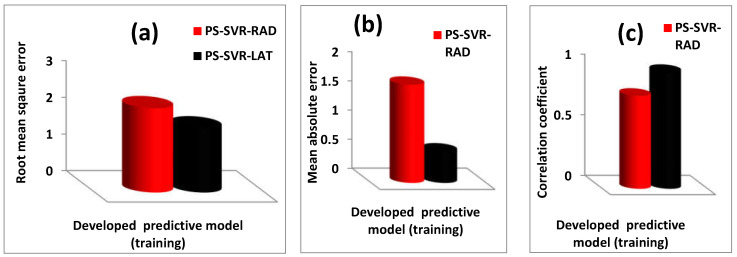

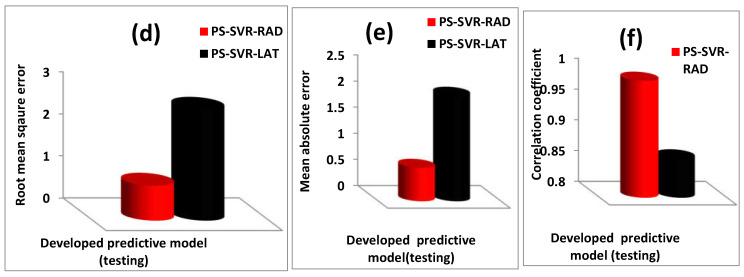
Performance evaluation and comparison for the developed PS-SVR-RAD and PS-SVR-LAT models.(**a**) comparison of the root mean square error of the developed model during training phase (**b**) comparison of the mean absolute error of the developed model during training phase (**c**) comparison of the correlation coefficient of the developed model during training phase (**d**) comparison of the root mean square error of the developed model during testing phase (**e**) comparison of the mean absolute error of the developed model during testing phase (**f**) comparison of the correlation coefficient of the developed model during testing phase.

A comparison of the performance of the developed PS-SVR-RAD and PS-SVR-LAT models is presented in Figure 4 using RMSE, MAE and CC performance metrics for the training and testing phases of model development. Figure 4a,b, respectively, presents the comparison of the performance of the developed PS-SVR-RAD and PS-SVR-LAT models during the training phases using RMSE and MAE metrics. The developed PS-SVR-RAD model outperforms the PS-SVR-LAT model with performance superiority of 31.60 and 67.92%, as presented in Figure 4a,b, respectively. A similar comparison on the basis of CC shows a performance improvement of 25.65%, as presented in Figure 4c. Testing stage performance comparison is presented in Figure 4d–f on the basis of RMSE, MAE and CC, respectively. The developed PS-SVR-LAT model shows a superior performance of 68.43, 68.22 and 12.73% on the basis of RMSE, MAE and CC, respectively, over the PS-SVR-RAD model. The performances of each of the developed models with their corresponding evaluation parameters are presented in Table 2 for the training and testing phase of model development. Considering the entire data points (training and testing sets), the developed PS-SVR-RAD model performs better than the PS-SVR-LAT model, with performance improvement of 15.28, 7.62 and 72.12%, on the basis of RMSE, CC and MAE, respectively, using the root mean square error (RMSE), coefficient of correlation (CC) and mean absolute error as performance measuring criteria.

### 4.3. Comparison of the Results of Developed Hybrid Models with Experimental Values

A comparison of the outcomes of the developed hybrid PS-SVR-LAT and PS-SVR-RAD models with the experimental values is presented in Table 3. The table also presents the absolute error between the estimated superconducting critical temperature (Tc) and measured values. The results of the developed PS-SVR-RAD model agree well with the measured values, while only a few compounds show little deviation. The results of the developed PS-SVR-LAT model also agree well with the measured values. The intrinsic properties of the SVR-based algorithm with excellent modeling strength, even with a limited number of data points, enhance the predictive capacity of the developed hybrid PS-SVR models.

## 5. Conclusions

The superconducting transition temperatures of 122 families of iron-based superconductors are modeled in this work using hybridization of particle swarm optimization algorithm with support vector regression. The developed PS-SVR-LAT model employs the lattice parameters emanated from tetragonal to orthorhombic structural transformation as descriptors while the ionic radii, as well as the concentration of the incorporated dopants, serve as the descriptors to the developed PS-SVR-RAD model. The developed PS-SVR-RAD model outperforms the PS-SVR-LAT model with performance superiority of 31.60 and 67.92% using RMSE and MAE for performance comparison for the training phase of model development. The developed PS-SVR-RAD model can easily model the 122-type of iron-based superconductors with four different incorporated dopants. The performance superiority, flexibility to accommodate multiple dopants at the same time and ease of implementation (since the ionic radii descriptors are easily fetched) of the developed PS-SVR-RAD model are of immense significance in effectively tailoring the superconducting critical temperature of a doped 122-type iron-based superconductor for specific and desired applications.

## Figures and Tables

**Table 1:** Global solutions for support vector regression (SVR) parameters obtained using particle swarm optimization (PSO). (PS = particle sarm, LAT = lattice, RAD = radii).

Hyper-Parameter	PS-SVR-LAT	PS-SVR-RAD
Epsilon	0.8	0.0001
Mapping function	Gaussian	Gaussian
Population size	50	10
Lambda hyper-parameter	E-7	E-7
Penalty factor	128.9911	9.8887
Kernel parameter	0.7263	0.009

**Table 2:** Comparison of the model performance evaluation parameters. (CC = correlation coefficient, RMSE = root mean square error, MAE = mean absolute error).

Stage	Training			Testing		
Model	CC	RMSE	MAE	CC	RMSE	MAE
PS-SVR-RAD	0.768	2.2987	1.6868	0.9897	0.8329	0.6426
PS-SVR-LAT	0.965	1.7468	0.5411	0.8637	2.6389	2.0222

**Table 3:** Outcomes of the developed hybrid models and their comparison with the measured values [29,30,31].

Iron-Based Compound	Tc (K)	PS-SVR-RAD (K)	Absolute Error	Iron-Based Compound	Tc (K)	PS-SVR-LAT (K)	Absolute Error
KFe_2_Se_2_	30.00	30.00	0.00	KFe_2_Se_2_	30.00	30.32	0.32
K_0.86_Fe_2_Se_1.82_	31.00	31.00	0.00	K_0.86_Fe_2_Se_1.82_	31.00	30.20	0.80
K_0.8_Fe_2_Se_1.96_	29.50	29.50	0.00	K_0.8_Fe_2_Se_1.96_	29.50	31.37	1.87
K_0.86_Fe_1.84_Se_2.02_	30.00	30.00	0.00	* K_0.8_Fe_2_Se_2_	33.00	31.48	1.52
K_0.8_Fe_1.6_Se_2_	32.00	32.00	0.00	K_2_Fe_4_Se_5_	32.00	32.23	0.23
Rb_0.78_Fe_2_Se_1.78_	32.00	32.00	0.00	K_0.86_Fe_1.84_Se_2.02_	30.00	30.80	0.80
Rb_0.8_Fe_2_Se_2_	31.00	27.61	3.39	* K_0.8_Fe_1.6_Se_2_	32.00	31.40	0.60
* Cs_0.86_Fe_1.66_Se_2_	30.00	30.00	0.00	Rb_0.78_Fe_2_Se_1.78_	32.00	31.20	0.80
Cs_0.8_Fe_2_Se_1.96_	27.00	27.00	0.00	Rb_2_Fe_4_Se_5_	32.00	32.80	0.80
TlFe_1.7_Se_2_	22.50	22.50	0.00	Cs_0.86_Fe_1.66_Se_2_	30.00	27.81	2.19
* Tl_0.75_K_0.25_Fe_1.85_Se_2_	31.00	27.61	3.39	Cs_0.8_Fe_2_Se_1.96_	27.00	27.80	0.80
Tl_0.61_K_0.39_Fe_1.76_Se_2_	25.10	25.10	0.00	Cs_2_Fe_4_Se_5_	29.00	29.80	0.80
Tl_0.58_Rb_0.42_Fe_1.72_Se_2_	32.00	32.00	0.00	* Tl_0.75_K_0.25_Fe_1.85_Se_2_	31.00	30.83	0.17
* K_0.8_Fe_2_Se_1.4_S_0.4_	32.80	33.20	0.40	Tl_0.61_K_0.39_Fe_1.76_Se_2_	25.10	26.67	1.57
K_0.8_Fe_2_Se_1.6_S_0.4_	33.20	33.20	0.00	Tl_2_Fe_4_Se_5_	31.00	29.49	1.51
K_0.8_Fe_2_Se_1.2_S_0.8_	24.60	24.60	0.00	Tl_0.58_Rb_0.42_Fe_1.72_Se_2_	32.00	31.43	0.57
K_0.8_Fe_2_Se_0.8_S_1.2_	18.20	18.20	0.00	Rb_2_Fe_4_Se_5_	28.00	29.00	1.00
BaFe_1.9_Co_0.1_As_2_	19.00	19.00	0.00	K_0.8_Fe_2_Se_1.4_S_0.4_	32.80	28.30	4.50
BaFe_1.866_Co_0.134_As_2_	25.00	25.00	0.00	K_0.8_Fe_2_Se_1.6_S_0.4_	33.20	28.14	5.06
BaFe_1.81_Co_0.19_As_2_	19.00	19.00	0.00	* K_0.8_Fe_2_Se_1.2_S_0.8_	24.60	24.32	0.28
BaFe_1.85_Co_0.15_As_2_	25.00	25.00	0.00	K_0.8_Fe_2_Se_0.8_S_1.2_	18.20	23.26	5.06
* BaFe_1.85_Co_0.15_As_2_	25.50	25.00	0.50				
BaFe_1.8_Co_0.2_As_2_	24.50	24.50	0.00				
BaFe_1.85_Co_0.15_As_2_	25.30	25.00	0.30				
BaFe_1.84_Co_0.16_As_2_	22.00	22.00	0.00				
BaFe_1.9_Ni_0.1_As_2_	20.00	20.00	0.00				
BaFe_1.91_Ni_0.09_As_2_	18.00	18.00	0.00				
* Ba_0.6_K_0.4_Fe_2_As_2_	38.00	37.50	0.50				
Ba_0.6_K_0.4_Fe_2_As_2_	35.00	37.50	2.50				
Ba_0.6_K_0.4_Fe_2_As_2_	37.50	37.50	0.00				
Ba_0.67_K_0.33_Fe_2_As_2_	38.00	37.55	0.45				
Ba_0.65_K_0.35_Fe_2_As_2_	34.00	34.00	0.00				
Ba_0.55_K_0.45_Fe_2_As_2_	23.00	27.61	4.61				
Ba_0.3_K_0.7_Fe_2_As_2_	22.00	22.00	0.00				
Ba_0.1_K_0.9_Fe_2_As_2_	9.00	17.72	8.72				
K_0.8_Fe_2_As_2_	31.00	31.00	0.00				
* Cs_0.8_Fe_2_Se_2_	30.00	27.61	2.39				
Tl_0.63_K_0.37_Fe_1.78_Se_2_	29.00	29.00	0.00				
Ba_0.55_K_0.45_Fe_2_As_2_	30.00	27.61	2.39				
* Ba_0.87_La_0.13_Fe_2_As_2_	22.50	27.61	5.11				
K_0.8_Fe_1.78_Se_2_	32.00	32.00	0.00				
Rb_0.8_Fe_1.6_Se_2_	32.40	32.40	0.00				

* test set of data.

## Data Availability

Data is available in the Supplementary Materials and in Table 3 of Section 4.2.

## Supplementary Materials

The following are available online at https://www.mdpi.com/article/10.3390/ma14164604/s1. The supplementary material contains the set of data employed in building the presented models.
